# The Time Constant of Attentional Control: Short, Medium and Long (Infinite?)

**DOI:** 10.5334/joc.24

**Published:** 2018-05-14

**Authors:** Leonardo Chelazzi, Elisa Santandrea

**Affiliations:** 1 Department of Neuroscience, Biomedicine and Movement Sciences, University of Verona, Verona, IT; 2 National Institute of Neuroscience, Verona, IT

**Keywords:** Attentional control, Experience-dependent attentional control, Priority maps of space

The review article by Jan Theeuwes (2018) mainly focuses on the distinction between top-down attention, which is assumed to be slow, effortful and controlled, and biases in selection stemming from ***selection history,*** which are assumed to be fast, effortless and automatic (and typically implicit). This distinction does not imply no role for purely salience-driven, or bottom-up, attention, but the latter topic is less crucial here. The term selection history alludes to the fact that the history of selection episodes, both from the recent and more distant past, can elicit lingering selection biases that are unrelated to top-down control or to the physical salience of objects. One further claim is made in the review, namely, that signals pertaining to the current goals of the individual (top-down), the intrinsic stimulus salience (bottom-up) and selection history biases all converge onto the same priority map(s) of space, wherein the compound signal will determine within a winner-take-all architecture which particular stimulus/location will be selected first.

In our commentary to this interesting and stimulating review paper, we want to draw attention onto three specific points that might fuel discussion, as briefly outlined below.

First, we propose that what the author refers to as “selection history” might be more appropriately defined as ***experience-dependent attentional control,*** since previous encounters with a given object and/or a given location must not have involved specific attentional operations (i.e., selection or suppression) to exert an influence on ongoing attentional deployment. In line with this notion, some of the studies carried out over recent years to study the influence of reward on attentional control (for relevant reviews, see Anderson, 2016; Bourgeois et al., 2016; Chelazzi et al., 2013; Failing and Theeuwes, in press) employed task paradigms where stimulus-reward associations were created during an initial learning phase devoid of any obvious selection component (e.g., Rutherford et al., 2010). In this context, it will not be possible to ascribe the observed effects to selection history; rather one might hypothesize that they derive from a changed status of certain stimuli in their brain representation. This raises the interesting possibility that experience might be so effective in inducing plasticity in the brain to even shape the basic architecture of visual processing, at least in the form of representations in primary sensory cortices (e.g., Shuler & Bear, 2006; Stănişor et al., 2013), thus mimicking the neural signatures of true bottom-up effects (see below), as the author himself suggests in the final part of his review, although from a phenomenological, rather than neurophysiological, perspective.

Second, we think that a neurophysiological perspective might be of help to better define the different forms of attentional control the author illustrates. Within this perspective, we propose to define: i) ***Bottom-up attentional control*** as a semi-stable, hard-wired property of the human brain, directly reflecting basic computation principles such as local contrast or center-surround antagonism (e.g., Angelucci et al., 2017), that for example makes the individual naturally reactive to items that differ from their surroundings (e.g., Treisman & Gelade, 1980; Yantis & Jonides, 1984; Posner & Petersen, 1990; Theeuwes, 1992); ii) ***Top-down attentional control*** as a flexible, goal-oriented and intentional form of attention, which is implemented through the dynamic online reconfiguration of relevant brain networks, likely requiring time-consuming (back) propagation of signals from control regions to target areas responsible for visual processing of the retinal input (Baluch & Itti, 2011); and, finally, iii) ***Experience-dependent attentional control*** as an instrumental tool for maximizing fitness to the environment, which is implemented through (more or less dramatic and durable) plastic changes in critical neural substrates.

Third, we would like to make some comments regarding the schematic illustration shown in Figure 1 of the review article by Theeuwes, depicting the integration of three sources of bias within the same priority map(s) of space. Our reflection on this point is threefold. On the one hand, we remark the possibility that different sources for attentional control, or biasing signals, rather than being merged through a simple computation (e.g., linear summation), as suggested by the author, might be weighted differently depending on the given context and task demands. For instance, one might conceive the possibility that, depending on the given circumstances, one or the other source will exert greater influence on the priority map, therefore being the primary determinant of attentional deployment; in other words, as recently suggested by Todd and Manaligod, a context-dependent weighting of different biasing signals for AC might be at place (Todd & Manaligod, 2017). On the other hand, it is still an open question whether a “minus sign”, i.e., the possible existence of de-prioritization signals, only pertains to experience-dependent forms of attentional control, as suggested here, or might be also implemented through top-down attention (and perhaps even through bottom-up factors). Yet another, and final, consideration relates to the possibility that multiple priority maps do exist in the brain, each representing priority of objects and locations in the visual space in relation to a specific motor output of the organism, i.e., as linked to the relevant effector for the given behavioral outcome (e.g., Rizzolatti et al., 1987; Berman & Colby, 2009). In line with this notion, an independent allocation of attentional resources to targets of eye and hand movements was demonstrated at the service of non-coordinated gaze and reaching actions (Jonikaitis & Deubel, 2011). Further evidence bearing on all these issues is required, such that the refinement of the notion of priority map(s) will likely be a very fertile area of investigation in the years to come.
